# What Is the Role of Weight (Dis)Satisfaction, Acculturative Stress, and Social Networks in BMI? An Exploration Among in Mexican Immigrants in New York City

**DOI:** 10.3390/ijerph23010052

**Published:** 2025-12-31

**Authors:** Karen R. Flórez, Emma Gutierrez

**Affiliations:** Graduate School of Public Health and Health Policy, City University of New York, New York, NY 10027, USA

**Keywords:** weight (dis)satisfaction, acculturative stress, social influence hypothesis, social networks, obesity

## Abstract

**Highlights:**

**Public health relevance—How does this work relate to a public health issue?**

- Acculturative stress and weight dissatisfaction jointly contribute to higher BMI among Mexican immigrants, underscoring the importance of psychosocial determinants in shaping obesity risk.
- Perceptions of social network members’ body size significantly predict BMI, with individuals reporting more overweight/obese alters showing lower BMI—revealing a nuanced and culturally contextualized role of social norms in immigrant health.

**Public health significance—Why is this work of significance to public health?**

- Findings demonstrate that legal-status-related acculturative stress—rather than general acculturation—exerts a measurable impact on BMI, highlighting a structural stressor with biological and behavioral consequences.
- The inverse relationship between perceived network overweight and individual BMI challenges conventional “obesity contagion” assumptions, suggesting that networks may also serve as normative buffers promoting body acceptance and potentially mitigating stress-related weight gain.

**Public health implications—What are the key implications or messages for practitioners, policy makers and/or researchers in public health?**

- Obesity interventions for immigrant communities must address structural stressors, including immigration-related insecurity and discrimination, which amplify weight dissatisfaction and contribute to higher BMI.
- Public health strategies should leverage the protective potential of social networks, recognizing that culturally aligned, body-size-diverse networks may reduce weight-related distress and offer resilience against thin-ideal pressures.

**Abstract:**

Acculturation and social networks shape ideals of weight perception, given that the construct is steeped in cultural perceptions of beauty and norms. This study leverages social network data from New York City (n = 80_participants_; 1600_network members_) who were asked “Would you like to weigh more, less, or stay the same?” as well as questions regarding their acculturation stress levels and the perceived weight of social network members. Body Mass Index (BMI) was objectively measured. Regression models evaluated the association between BMI, weight satisfaction, and acculturation stress, net of sociodemographic variables, weight loss attempts, and health behaviors. Those who were dissatisfied with their weight and experienced high acculturation stress had a significantly higher BMI (β = 5.4, 2.8–8.1, *p* < 0.001) in the fully adjusted model. However, with every 25% increase in the perception of network members with obesity/overweight, there was a significant decrease in individual BMI. No other social network variables were significantly associated with BMI. More research is needed among Latinos/as across the acculturative stress spectrum to fully understand how social norms regarding weight affect both social networks and individuals, as well as how these might be influenced by cross-cultural differences between US and Latino/a norms regarding ideal weight perceptions.

## 1. Introduction

Obesity is an established risk factor for chronic conditions that disproportionately burden Latinos/as of Mexican descent, including type 2 diabetes [1]. These risks can be modified through healthy behaviors, such as diet and physical activity. However, nationally representative studies show that for Mexican Americans, healthful diet-related behaviors tend to decline with increasing acculturation, measured by years in the United States or English-language adoption [2]. For instance, English-dominant Latinos/as report more frequent consumption of restaurant and convenience foods than their Spanish-dominant peers [3]. The prevalence of obesity mirrors these trends: U.S.-born Mexican Americans have higher rates of obesity than Mexican immigrants and the general U.S. population [4]. However, greater acculturation is also associated with higher levels of leisure-time physical activity [5]. These mixed findings underscore that acculturation affects health through multiple, sometimes opposing, pathways that are not yet well understood in the literature.

One mechanism that may clarify these patterns is the influence of social networks. Social networks shape obesity, diet, and physical activity through social support, access to resources, and the diffusion of norms [6,7,8,9]. Individuals are more likely to have obesity if their close contacts do, a phenomenon attributed to shared norms and behavioral contagion [10]. Studies have shown that social norms of eating, family and peer influences, and perceived support can affect food choices and overall energy balance [11,12,13]. However, the direction of these influences can vary: networks may reinforce either healthy or unhealthy eating patterns depending on group norms [14]. Among Latinos/as, whose networks often include both co-ethnic and U.S.-born members, the acculturation process may shift social norms around body image and food; however, this dynamic has been underexplored.

Acculturation refers to the process through which immigrants adopt the norms, values, and behaviors of the dominant culture while maintaining varying degrees of connection to their heritage culture [5]. Cultural ideals about body weight and beauty are among the domains most affected by acculturation. Within Mexican and broader Latino/a cultures, fuller body shapes are often viewed as healthy, maternal, and attractive—values that can buffer against body dissatisfaction and extreme dieting behaviors [15,16]. Comparative research from Latin America provides further evidence that among women, having excess weight meant being “full of life” and outlines an expectation of weight gain from señoritas (single) to señoras (married) [17,18]. In contrast, U.S. cultural norms valorize thinness, particularly among women, and equate slimness with discipline and success [19]. As immigrants are exposed to these ideals, acculturation can generate cognitive dissonance between heritage-based body norms and U.S. standards. How individuals negotiate these conflicting messages may shape both their perceived body satisfaction and their actual body mass index (BMI).

Importantly, acculturation does not occur without strain and frameworks like segmented assimilation, intersectionality, and stress-response models highlight the structural conditions under which immigrant adaptation unfolds. For example, racialization and discrimination serve as major barriers to integration into the American mainstream, and these constraints are compounded by broader economic shifts that limit avenues for intergenerational mobility [20]. Furthermore, scholars using an intersectionality lens to study immigrant health trajectories have highlighted the need to understand changes in immigrant health within the macro-level hierarchical forces that simultaneously shape outcomes such as class and gender-based systems [21]. Indeed, when Latinos are ascribed a lower status in this hierarchy, there is mounting evidence that their health suffers through stress-response processes [22], which include differences across gender and nativity [23,24].

Indeed, the stresses associated with adapting to a new social environment—language barriers, discrimination, and loss of social support—are captured by a related-but-distinct concept of acculturative stress [25]. Acculturative stress highlights the psychological costs of integration, especially when immigrants experience pressure to conform to dominant norms or encounter stigma due to language, documentation status, or ethnicity [26]. These stressors have been linked to poorer self-rated health, disordered eating, and sedentary behavior [27]. Moreover, emotional responses to discrimination (e.g., anger or hopelessness) are associated with biological processes that promote adiposity [28]. Thus, while acculturation itself may introduce both risks and resources, acculturative stress represents the strain that can translate social and structural disadvantages into adverse health outcomes, including obesity.

Social networks can either mitigate or amplify stress processes. Supportive ties, particularly co-ethnic networks, can buffer acculturative stress by providing emotional and instrumental support, while more assimilated or diverse networks may expose individuals to dominant weight norms and reinforce the pressure to lose weight or achieve thinness. Conversely, networks characterized by high perceived obesity or overweight status may normalize higher BMI and reduce body dissatisfaction. Understanding how these structural and compositional network features interact with acculturative stress to influence BMI remains a critical gap.

Taken together, existing evidence suggests that the BMI of Latino/a immigrants reflects the intersection of acculturation-related cultural shifts, the stresses of adaptation, and the social environments in which these processes unfold. However, few studies have simultaneously examined these mechanisms. To address this gap, we drew on social network data from a community sample of Mexican-heritage adults in New York City to explore how legal-status-related acculturative stress and social network characteristics jointly shape body perception and BMI.

We address the following research questions and hypotheses.

**RQ1.**  *Is body (dis)satisfaction associated with objectively measured BMI, net of sociodemographic factors and weight loss attempts?*

**Hypothesis** **1.** *Individuals who report greater body dissatisfaction will have a higher BMI, reflecting a misalignment between internalized body ideals and actual body weight.*

**RQ2.**  *Does legal-status-related acculturative stress moderate the association between body (dis)satisfaction and BMI?*

**Hypothesis** **2.** *The positive association between body dissatisfaction and BMI will be stronger among individuals experiencing higher levels of acculturative stress, as stress may exacerbate emotional or behavioral responses (e.g., disordered eating and reduced activity) that contribute to weight gain.*

**RQ3.**  *Are social network features—structural (e.g., diversity, transitivity) and compositional (e.g., proportion of alters perceived as overweight/obese)—associated with BMI?*

**Hypothesis** **3a.** *Individuals embedded in networks with a higher proportion of alters perceived as overweight/obese will have lower body dissatisfaction and, consequently, a lower BMI due to normative acceptance of diverse body sizes.*

**Hypothesis** **3b.** *Conversely, more diverse or less cohesive networks may expose individuals to dominant U.S. body norms and thus be associated with higher body dissatisfaction and BMI.*

By reframing acculturation to encompass both stress and social context, this study contributes to a more integrated understanding of how cultural and structural forces jointly shape body image and obesity risk among Latino/a immigrants in the United States. We also hypothesized that chronic stress related to legal status discrimination is on the causal pathway to BMI by altering eating behaviors and metabolic functioning. Simultaneously, immigrants navigate competing cultural messages regarding ideal body size, and social networks transmit and reinforce these norms. BMI may therefore reflect the combined effects of stress-induced physiological and behavioral responses and network-based normative influences on weight.

## 2. Materials and Methods

Data were obtained from the Social Network of Mexican Americans (SNMA) study [29]. The original study was designed to investigate how the social networks of Mexican immigrants living in NYC influenced and were related to diabetes-related health behaviors. Given the importance of weight in diabetes self-management, this analysis leveraged more nuanced measures, such as weight (dis)satisfaction. Participants (N = 81 egos; 1600 alters) were recruited through a Catholic church in the Bronx, New York. The eligibility criteria for participation included being at least 18 years of age and self-identifying as Chicano, Mexican, or Mexican American. Interviews were conducted in person between January and June 2019 by trained bilingual research assistants. The network portion of the interview was administered using EgoWeb [30], using standard elicitation procedures for social networks, in which they were asked to name the 20 most important persons in their lives (called “alters”), describe how they knew them, and indicate whether they had been in contact with them (face-to-face, by phone call, text/phone app message, email, or social media) in the last six months prior to the interview. Participants were then asked about their alters’ demographics, frequency of contact, relevant health characteristics, and whether their personal network was densely or sparsely connected and the extent to which the alters knew each other. Additional demographic and individual-level characteristics, including health-related information, were collected using the Qualtrics platform [31]. Finally, anthropometric measurements of height and weight were collected by the same trained bilingual research assistant. All study procedures were approved by the City University of New York Human Subjects Institutional Review Board (IRB File No. 2018-1081)

### 2.1. Dependent Variable

**BMI:** Interviewers were trained to measure height using a portable height-measuring board by SECA, Germany. Height was recorded to the nearest one-eighth of an inch, with any adjustments noted (e.g., for shoes or hair ornaments that the respondent chose not to remove). Respondents’ weights were measured using the SECA Robusta 813 digital scale. BMI was derived using the standard formula of weight (kg) divided by height squared (m^2^).

### 2.2. Independent Variable: Individual-Level and Joint Effects

**Weight Satisfaction.** Participants reported whether they would like to “weigh more,” “stay the same,” or “weigh less” using a question from the National Health and Nutrition Survey [32]. Given the small number in the “weigh more” category (n = 6), we present all three categories in descriptive analyses but collapsed them into a dichotomous indicator of “satisfied” versus “dissatisfied” for multivariable models to preserve statistical power. Therefore, the results should be interpreted as reflecting general dissatisfaction rather than the specific direction of desired change.

**Acculturation proxies** the **Marin Scale.** Acculturation was assessed through three distinct domains: language use, media, and social relations [33]. The overall score was computed by summing the scores on all scale items and dividing this by the number of items on the scale (or the number of items that the participant responded to). The scores ranged from 1 to 5, with higher scores indicating greater acculturation to the U.S. In our sample, the coefficient alpha for the Marin Scale was (α = 0.90), indicating excellent reliability. **Age at migration** to the U.S. Respondents reported their age when they moved to the U.S.

**Weight satisfaction × Acculturative Stress.** We operationalized acculturative stress for the joint-effects analysis using a single item capturing worries about legal status, selected a priori to isolate a structural stressor that was theorized to moderate the association between weight (dis)satisfaction and BMI. This choice enhanced the interpretability of the four joint categories and avoided arbitrary cut points on a multi-item score. Specifically, (a) satisfied/low acculturation stress (reference, since it is the stratum with the hypothesized lowest risk), (b) satisfied/high acculturation stress, (c) dissatisfied/low acculturation stress, and (d) dissatisfied/high acculturation stress). Because interaction estimates are sensitive to measurement error, we favored a targeted indicator for the moderator in our modest sample size.

### 2.3. Social Network-Level Variables

**Figure 1:**
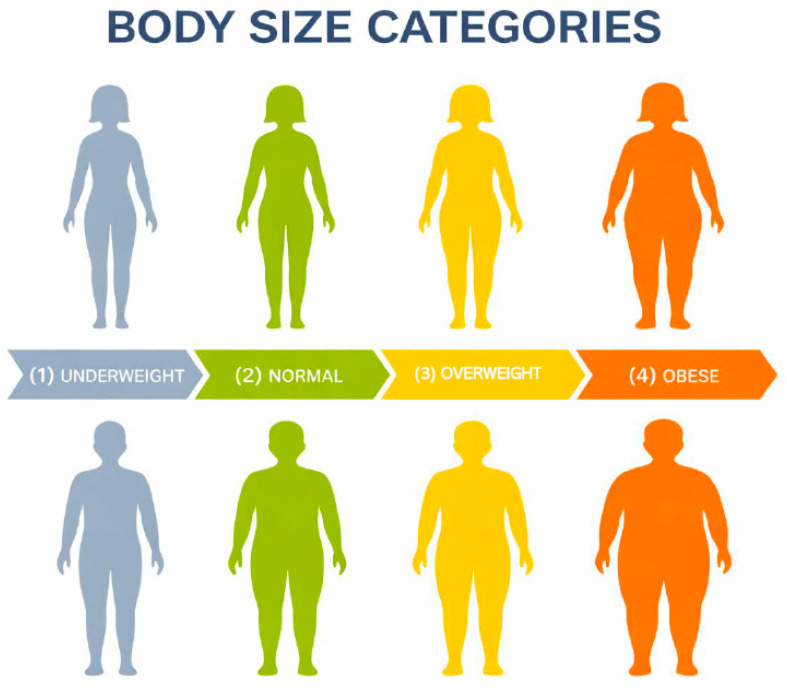
Figure Rating Scale.

**Percentage of social network perceived with overweight or obesity and other network indicators:** This measure was calculated based on the Stunkard scale, which includes silhouetted illustrations of people that gradually increase in size that correspond to (1) underweight, (2) normal weight (3) overweight, and (4) obese, with separate figures for women and men (see Figure 1) [34]. The Figure Rating Scale has been used in research with Latino populations of Mexican descent [35], has good test–retest reliability [36], and has established validity among immigrant Latinos [37]. Participants were asked “Which of these images best reflects Person X’s current appearance” and selected one image for each alter. The number of alters perceived by the ego with overweight or obesity was divided by the total number of alters in the network (n = 20) to calculate the percentage of the ego’s social network that they perceived as overweight or obese. This was calculated for each participant separately. While prior applications have focused primarily on egos’ self-assessments, our use of the scale to characterize alters is a natural extension of this approach. Perceptions of others’ body size are central to the processes of social comparison and normative influence—two mechanisms widely recognized in social network and health research as shaping health behaviors and outcomes. Thus, while objective measures of alters’ BMI were not feasible, the use of perceptual ratings is theoretically justified and empirically meaningful, as individuals’ perceptions of their social environment most directly influence their attitudes and behaviors.

In addition to these person-specific attributes from alters that characterize who is in the network based on weight perceptions, our data contain information on the structural properties or the relational characteristics between network members. Specifically, we calculated density (which represents the proportion of ties among alters and reflects how tightly knit a participant’s network is, transitivity (measures clustering—the likelihood that an alter’s contacts also know one another—indicating cohesive subgroups that may reinforce shared norms, and constraint (captures the degree of redundancy in the network; higher values suggest that alters provide overlapping information or influence) for each participant [38].

**Covariates**: Sociodemographic variables included age (continuous), sex (male, female), household yearly income (≤$30 K, >$30 K, or missing), education (less than a high school diploma/general educational development [HS, HS diploma/GED or greater]), relationship status (married/living with a partner, or else), and attempted to lose weight in the past 12 months (yes/no). For health behaviors, we used the total Healthy Eating Index (HEI) [39], which was derived from two 24 h dietary recalls using the ASA-24 tool [40]. This yields a score of 0–100, with higher scores indicating better adherence to the USDA dietary guidelines [39]. PA was measured using the validated International Physical Activity Questionnaire (IPAQ) Short Form (IPAQ-SF) [41]. The scores were derived using a standard algorithm, with higher scores indicating increased physical activity.

### 2.4. Statistical Analysis

For the present analysis, we excluded one participant who only spoke English, given the focus on acculturation, for a total analytic sample of 80 egos and 1600 social network members. Descriptive statistics were calculated for the entire sample, and chi-square and *t*-tests were used to calculate how these differed according to weight satisfaction. Furthermore, means and SDs were generated for all continuous data (e.g., age, HEI, and IPAQ), while categorical data (e.g., sex, income, education, and marital status) were reported as frequencies and percentages. Bivariate Pearson’s *r* correlation coefficients were calculated to characterize the degree to which weight-related and acculturation factors were related to each other and to BMI. We tested the joint effect of weight satisfaction and acculturation stress using a 4-level categorization scheme noted in the measures section, using the stratum with the lowest risk as the reference category, following best practices [42]. This model, as well as all marginal effects, controlled for sociodemographic variables (age, sex, marriage, education, and income), acculturation (Marin Scale and age at migration), weight loss attempts, proportion of obese/overweight network members, and health behaviors (total Healthy Eating Index and IPAQ score). Finally, using the margins and contrast functions in STATA, we tested the predicted probability of BMI change as a function of the proportion of the network perceived with overweight and obesity by the ego at 25% intervals, which aligns with prior social network and obesity research testing non-linear trends and threshold effects. The significance level was set at *p* < 0.05. All analyses were conducted using STATA version 15.1 [43].

### 2.5. Diagnostic Procedures

We employed a hierarchical regression strategy to maximize power and minimize the risk of overfitting, given the modest sample size [14]. The initial models included only sociodemographic covariates and the primary study variable (weight dissatisfaction × acculturative stress). Subsequent models added acculturation proxies, weight loss attempts, health behavior variables, and network composition. This stepwise approach allowed us to assess the stability of the primary associations while limiting the number of predictors in the early iterations. To evaluate the model diagnostics, we examined the variance inflation factors (VIFs), residual plots, and influence statistics (Cook’s D and leverage) across the models. Specifically, the variance inflation factors (VIFs) across all models were low (range, 1.09–2.12), indicating the absence of multicollinearity. Cook’s distance values were well below the conventional threshold of 1.0 (max = 0.105), and studentized residuals fell within ±3 (range −2.88 to 2.70), suggesting the absence of problematic outliers. While a small number of cases exhibited leverage slightly above 0.20 (max = 0.351), they did not unduly influence the results, as the significance and magnitude of the main effects remained stable across the hierarchical models. Together, these diagnostics indicated that our models were not overfitted and that the observed associations were strong.

## 3. Results

Table 1 presents the sociodemographic and behavioral characteristics of the sample (N = 80) by weight satisfaction status. The mean age was 43 years (SE = 1.3), with most participants identifying as female (70%) and reporting annual household incomes below $30,000 (72.5%). Over half (58.7%) had less than a high-school education, and 60% were married or living with a partner. Compared to those satisfied with their weight, participants who wanted to lose weight were more likely to be married or cohabiting (65.5% vs. 16.7%, *p* = 0.009). No other sociodemographic differences were statistically significant. Mean Healthy Eating Index scores were similar across groups, averaging 57.7 (SE = 1.4) for the full sample, with slightly higher adherence among those wishing to gain weight (61.0 ± 4.9). Physical activity levels, as measured by the IPAQ, were modest overall (M = 1.88 ± 0.09), with higher scores observed among participants desiring to gain weight (2.3 ± 0.19) than among those wanting to lose weight (1.7 ± 0.10).

Table 2 summarizes the correlations among weight-related, acculturation, and social network variables. BMI was positively correlated with weight dissatisfaction (r = 0.49, *p* < 0.05), weight-loss attempts (r = 0.31, *p* < 0.05), overall acculturative stress (r = 0.32, *p* < 0.05), and experiences of being asked about their legal status (r = 0.36, *p* < 0.05). Conversely, BMI was inversely correlated with general acculturation (r = −0.26, *p* < 0.01) and with the proportion of network members perceived as overweight or obese (r = −0.32, *p* < 0.05). These bivariate patterns provided initial support for RQ1 (weight dissatisfaction associated with higher BMI) and RQ2 (acculturative stress positively associated with BMI), while raising new questions about RQ3, given the unexpected inverse network relationship.

**RQ1: Association Between Weight Dissatisfaction and BMI**

Regression models (Table 3) indicated that, after controlling for sociodemographic factors and health behaviors, individuals dissatisfied with their weight had a significantly higher BMI than those satisfied (β = 2.9, 95% CI = 1.6–5.6, *p* < 0.05). This finding aligns with **Hypothesis 1**, supporting the idea that greater weight dissatisfaction corresponds to a higher objectively measured BMI.

**RQ2: Moderating Role of Acculturative Stress**

Next, we examined whether acculturative stress modified the association between weight dissatisfaction and BMI. Among individuals reporting **high acculturative stress**, weight dissatisfaction was strongly associated with a higher BMI (β = 5.4, 95% CI = 2.8–8.1, *p* < 0.001). Among those with **low stress**, the association persisted but was weaker (β = 2.9, 95% CI = 1.6–5.6, *p* < 0.05). The interaction term confirmed a significant synergistic effect (β = 4.9, 95% CI = 3.4–9.5, *p* = 0.03), indicating that dissatisfaction was more strongly linked to elevated BMI under conditions of higher acculturative stress. These results support **Hypothesis 2**, suggesting that the psychological strain of acculturative stress may exacerbate the emotional and behavioral pathways (e.g., emotional eating and reduced self-regulation) through which body dissatisfaction contributes to a higher BMI.

**RQ3: Social Network Composition and BMI**

**Figure 2:**
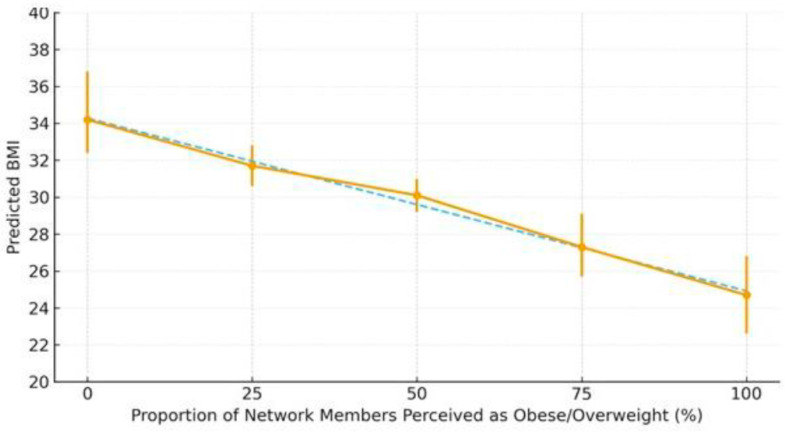
Predictive margins of the association between weight (dis)satisfaction, acculcurative stress, and BMI by the proportion of network members perceived as obese/overweight. Notes: Points show predictive margins with 95% CIs. X-axis reflects the share of network members perceived as obese/overweight. Y-axis shows predicted BMI.

Figure 2 shows the predicted BMI values by the proportion of network members perceived as overweight/obese, adjusting for the joint effects of weight dissatisfaction and acculturative stress. Among participants who reported both high stress and dissatisfaction, the predicted BMI was highest when few or no network members were perceived as overweight. As the proportion of overweight network members increased, the predicted BMI steadily declined (*p* < 0.05 for marginal effect). No other network structural features (e.g., transitivity and diversity) were significant.

## 4. Discussion

This study sheds new light on how psychosocial and social network factors intersect to influence obesity risk among Mexican immigrants in New York City, a population that continues to bear the brunt of multiple emerging public health challenges, including chronic stress and discrimination. Our findings show that acculturative stress, not general acculturation, amplifies the association between weight dissatisfaction and a higher BMI. This distinction is crucial: while previous studies have often focused on cultural adaptation or exposure to Western ideals [44], our data suggest that structural and legal anxieties—particularly fears surrounding immigration status—constitute an underrecognized form of chronic stress that may have both psychological and physiological consequences.

The joint effect of high acculturative stress and weight dissatisfaction on BMI indicates that these psychosocial stressors do not operate in isolation. Rather, they may act synergistically, compounding threats to self-worth and belonging, and triggering maladaptive coping responses, such as emotional eating and sedentary behaviors. These findings align with prior evidence linking acculturative stress to body image concerns among Latina women [45] and extend this literature by demonstrating measurable effects on BMI. Moreover, our focus on legal-status-related worries captures a structural stressor that is particularly salient for Latino immigrants, given how anti-immigrant sentiment and precarious labor conditions have intensified [46,47].

Interpreting these results through a biopsychosocial lens suggests that chronic exposure to exclusionary environments may lead to the embodiment of stress in the form of increased adiposity [28]. This process, well documented in other racialized groups, remains understudied in immigrant populations despite growing evidence of stress-related biological dysregulation [48]. This implies that BMI may serve not only as a marker of nutrition and behavior but also as a biomarker of structural vulnerability.

Because egocentric network designs measure only the respondent’s direct ties and selected alter–alter relationships, they do not capture the full topology of the larger network system [49]. This partial structural visibility reduces the variability in metrics such as density, transitivity, and constraint, which may help explain the absence of associations with BMI in this study. Our social network findings regarding alter attributes offer a more complex perspective. Contrary to the prevailing “obesity contagion” models [10] which informed Hypothesis 3a, participants with a higher proportion of network members perceived as overweight or obese had a lower BMI. This inverse association may indicate a normative buffering effect, wherein networks of individuals with diverse body sizes foster acceptance and reduce the internalization of stigmatizing ideals. We draw from research from social psychology and the tenets of social comparison theory [15], which predicts that people compare themselves to others around them (including peers) as well as the qualitative evidence that Mexican women engage in this type of social comparison regarding their weight [17]. It is also possible that shared experiences with chronic diseases within Latino networks encourage mutual vigilance around diet and health behaviors, particularly in communities with a high diabetes and cardiovascular disease burden [17]. However, measurement bias and reverse causality remain plausible, especially since participants were recruited from a single church community and they may share similar socioeconomic constraints, occupational exposures, or health challenges that shape both their network composition and their weight-related experience (e.g., clustering). Given research on self-perceptions of weight among Latinos [50], individuals with a lower BMI may systematically overestimate the prevalence of overweight among alters, whereas individuals with a higher BMI may underestimate it. This perceptual variability could help explain the observed inverse association between perceived network overweight and respondents’ own BMI, independent of any true normative influence.

### Limitations and Strengths

This study had several limitations. The cross-sectional design precludes causal inference, and network data were based on ego perceptions rather than alter ’self-reports. However, these perceptions are meaningful, reflecting how individuals interpret the social norms surrounding them. Although faith-based institutions remain central nodes of support for Mexican immigrant communities in NYC and beyond, recruitment through a single Catholic church in the Bronx may have introduced selection bias, yielding a sample that may be more female, socially connected, more religiously engaged, and more homogeneous in cultural and socioeconomic characteristics than the broader Mexican immigrant population in NYC. Such recruitment may also influence the network metrics observed, as shared congregational ties can increase cohesion and reduce network diversity, potentially shaping both perceived norms and stress processes. The findings should be interpreted as specific to this community context and not generalizable to all Mexican immigrants or Latino/a groups in different social contexts. Future studies should therefore draw from multiple recruitment sites—including workplaces, community organizations, and social service agencies—to capture a broader diversity across gender and network structures and to improve external validity.

An additional limitation concerns the dichotomization of weight dissatisfaction, where we did not distinguish between those wishing to weigh less and those wishing to weigh more (six participants in the latter group). Although small, this subgroup may have introduced residual confounding, as individuals who desire weight gain may differ from those wanting to lose weight in ways related to BMI, health behaviors, and cultural norms. Future studies should employ more nuanced measures to capture both the direction and motivation behind weight dissatisfaction, differentiating between esthetic, health-related, and psychological dimensions. Further, studies should incorporate self-perceived body size to explore how this may have differed from our objectively measured BMI and elucidate how weight-related norms may operate within networks.

Finally, another key limitation is the measurement of acculturative stress using a single item assessing worries about legal status. Although this structural stressor is theoretically salient and empirically linked to health among Latino immigrants, a single indicator cannot capture the multidimensional construct of acculturative stress and may increase the measurement error. Therefore, our measure reflects only one domain—legal and structural insecurity—while excluding stressors related to language, discrimination, or cultural conflict that measures such as the Hispanic Stress Inventory 2 tap for Latino immigrants (via 10 subscales) and US-born Latinos (via six subscales) [51]. This likely reduces construct validity and may attenuate the observed associations. Future studies should employ validated multi-item scales like the Hispanic Stress Inventory 2 to better differentiate domains of acculturative stress and their interactions with weight perceptions and network processes.

## 5. Conclusions

Our findings highlight acculturative stress—especially stress related to legal status—as an emerging public health concern for Latino immigrants. When combined with weight dissatisfaction, this stress manifests not only as psychological distress but also as increased BMI, pointing to the deep biological imprint of social exclusion. Addressing these intertwined processes requires a shift from individually oriented obesity interventions to strategies that mitigate the structural and psychosocial determinants of health, such as immigration insecurity, discrimination, and limited access to culturally safe care.

The observed inverse association between network overweight prevalence and BMI further suggests that social ties can act as protective buffers, underscoring the importance of community-based and relational approaches to migrant health promotion. Strengthening collective spaces, such as churches, community centers, and peer support networks, may help transform social norms and reduce body-related distress.

In the context of newly emerging public health issues such as mental stress and discrimination this study offers a timely contribution: it reframes obesity among immigrants not merely as an individual risk behavior but as a biopsychosocial response to structural adversity. Future research integrating longitudinal, biomarker, and agent-based modeling approaches will be essential for mapping these multilevel interactions and designing interventions that promote both health equity and psychosocial well-being in migrant communities.

## Figures and Tables

**Table 1:** Descriptive Statistics.

	Total N (%, M, SE)	Weight Less N (%, M, SE)	Stay the Same N (%, M, SE)	Weight More N (%, M, SE)
**Sociodemographic**				
Age, years	80 (43.1 ± 1.3)	58 (44.7 ± 1.3)	22 (38.9 ± 2.6)	6 (39 ± 5.3)
Sex, female	56 (70.0)	44 (75.9)	12 (54.6)	3 (50.0)
Income, <30K	58 (72.5)	45 (77.6)	13 (59.1)	5 (83.3)
Education, Less than H.S. Diploma	47 (58.7)	37 (63.8)	10 (45.5)	4 (66.7)
Married/Living with Partner	48 (60.0)	38 (65.5)	10 (45.5)	1(16.7)
**Weight-related Health Behaviors**				
Healthy Eating Index ^1^	80 (57.7 ± 1.36)	58 (56.8 ± 1.6)	22 (59.8 ± 2.4)	6 (61 ± 4.9)
IPAQ ^2^	80 (1.88 ± 0.09)	58 (1.7 ± 0.10)	22 (2.3± 0.19)	6 (2 ± 0.36)

^1^ higher score reflect more adherence to Dietary Guidelines for Americans; ^2^ higher score reflect higher levels of moderate or high physical activity.

**Table 2:** Correlations among study variables.

Variable	1	2	3	4	5	6	7	8	9	10
1. Body Mass Index (BMI)	—									
2. Weight dissatisfaction	0.49 *	—								
3. Weight loss attempted	0.31 *	0.15	—							
4. Legal status	0.36 *	0.34 *	0.13	—						
5. Marin acculturation scale	−0.09	−0.18	0.14	−0.12	—					
6. Age at migration	0.11	0.09	−0.06	−0.04	−0.02	—				
7. Perceived overweight/obesity in network	−0.32 *	−0.01	−0.07	0.08	0.01	0.08	—			
8. Network density	0.18	0.16	−0.01	0.10	−0.16	−0.10	−0.03	—		
9. Network transitivity	0.20	0.14	0.05	0.14	−0.06	−0.10	0.05	0.71 *	—	
10. Network constraint	0.04	0.02	−0.06	−0.03	−0.06	−0.10	0.08	0.66 *	0.26 *	—

Note. * *p* < 0.05. Values are Pearson’s correlation coefficients. Negative values indicate inverse associations.

**Table 3:** Joint effects of dissatisfaction and acculturative stress and BMI, (n = 80 participants; 1600 network members).

	Exposure = 0 (Satisfied)				Exposure = 1 (Dissatisfied)							
	β ^§^	Lower	Upper	*p*-Value	β ^§^	Lower	Upper	*p*-Value	β ^§^ of Weight Satisfaction Within Strata of Acculturative Stress			
0 = low acculturative stress	Ref	.	.	.	2.9	1.6	5.6	<0.05	2.7	1.1	3.1	0.04
1 = high acculturative stress	0.29	−3.8	4.4	0.88	5.4	2.8	8.1	<0.001	4.9	3.4	9.5	0.03
**β ^§^ of Acculturative Stress within Weight Satisfaction Strata**	1.1	−1.6	4.0	0.93	3.1	0.80	5.4	<0.001				

^§^ Adjusted for sociodemographic variables (age, sex, marriage, education, income, age at migration and acculturation), weight loss attempts, proportion obese/overweight network members, and health behaviors (total healthy eating index, IPAQ score).

## Data Availability

Data cannot be shared publicly because of personal identifiers. Data are available from the CUNY Data Access/Ethnics Committee (contact via https://sph.cuny.edu/research/human-research-protection-program/ (accessed on 30 October 2025) for researchers who meet the criteria for access to confidential data.

## Abbreviations

The following abbreviations are used in this manuscript:

NHANES	National Health and Nutrition Examination Survey
US	United States
BMI	Body Mass Index
HEI	Healthy Eating Index
IPAQ	International Physical Activity Questionnaire
ASA-24	The Automated Self-Administered 24-h
